# Kernel-density estimation and approximate Bayesian computation for flexible epidemiological model fitting in Python

**DOI:** 10.1016/j.epidem.2018.05.009

**Published:** 2018-12

**Authors:** Michael A. Irvine, T. Déirdre Hollingsworth

**Affiliations:** a Institute of Applied Mathematics, University of British Columbia, Vancouver, Canada; b Big Data Institute, Li Ka Shing Centre for Health Information and Discovery, Nuffield Department of Medicine, University of Oxford, Oxford, UK

**Keywords:** Approximate Bayesian computation, Individual-based model, Lymphatic filariasis, Model fitting, Python library

## Abstract

Fitting complex models to epidemiological data is a challenging problem: methodologies can be inaccessible to all but specialists, there may be challenges in adequately describing uncertainty in model fitting, the complex models may take a long time to run, and it can be difficult to fully capture the heterogeneity in the data. We develop an adaptive approximate Bayesian computation scheme to fit a variety of epidemiologically relevant data with minimal hyper-parameter tuning by using an adaptive tolerance scheme. We implement a novel kernel density estimation scheme to capture both dispersed and multi-dimensional data, and directly compare this technique to standard Bayesian approaches. We then apply the procedure to a complex individual-based simulation of lymphatic filariasis, a human parasitic disease. The procedure and examples are released alongside this article as an open access library, with examples to aid researchers to rapidly fit models to data. This demonstrates that an adaptive ABC scheme with a general summary and distance metric is capable of performing model fitting for a variety of epidemiological data. It also does not require significant theoretical background to use and can be made accessible to the diverse epidemiological research community.

## 1 Introduction

There is a trend towards greater realism using individual-based models within the ecological and epidemiological modelling community (Grimm et al., 2006, Bansal et al., 2007, DeAngelis and Grimm, 2014, Heesterbeek et al., 2015). The strength of this approach lies in its ability to directly address policy-relevant questions, however properly estimating model parameters and measuring uncertainty in fits is often problematic/challenging (Deardon et al., 2010; Grimm and Railsback, 2013). In addition, the data will often be highly heterogeneous making model fitting difficult. Examples of this in epidemiology include both human and animal parasitic infections, such a soil-transmitted helminths and nematodes, where the variance in egg counts can be bigger than the mean (Shaw et al., 1998, Elkins et al., 1986, Grenfell et al., 1990). Data may also come in the form of multivariate time-series, such as number of diagnoses in different disease stages or different age-categories or age/risk/disease stage-stratified prevalence (Hollingsworth et al., 2008; Pullan et al., 2014). These data can be challenging to fit as it can be noisy and may not be easily modelled by simple distributions.

Complex individual-based models will often have computationally-intractable likelihoods, or likelihoods that are not easily defined or applied to data. In such cases, approximate Bayesian computation (ABC) has been proposed as a valid approach to model fitting (Csilléry et al., 2010). ABC has primarily been used to fit approximately Gaussian or Poisson-type data in the context of epidemiology (McKinley et al., 2009, McKinley et al., 2014, Beaumont, 2010, Walker et al., 2010, Kypraios et al., 2016). Other data sources have been incorporated into model fitting using ABC, such a phylogenetic data (Tanaka et al., 2006, Luciani et al., 2009, Ratmann et al., 2012). It is often not clear what choice of summary statistic should be used and this is often domain specific, which can prevent these methods being applied elsewhere (Luciani et al., 2009, Marin et al., 2012).

Whilst these are general problems, they are of particular relevance in the calibration of complex individual-based models designed for policy-relevant questions. In this paper, we consider the case of lymphatic filariasis transmission. Lymphatic filariasis (LF) or elephantiasis is a neglected tropical disease, with over 40 million individuals displaying clinical manifestations of the disease, and with 53 countries requiring preventative chemotherapy. It is currently targeted for elimination as a public health problem by the World Health Organisation (WHO) by 2020 through the use of mass drug administration (MDA) (Rebollo and Bockarie., 2013, World Health Organization et al., 2011, Ottesen et al., 2008, Ottesen et al., 1997). As with many public health interventions, there is a certain amount of systematic non-adherence or heterogeneity in the use of interventions (Dyson et al., 2017). Coupled with this is the large amount of heterogeneity in exposure to infection across individuals. These complexities require that transmission models take into account the vector and parasite biology and human social factors (Irvine et al., 2015, Stolk et al., 2008, Chan et al., 1998). Due to the sparse nature of the data, parameter uncertainty in the fitted models must also be estimated if robust predictions are to be made (Singh and Michael, 2015). ABC then offers a strong alternative to other techniques for fitting complex individual-based models, which can also include uncertainty in the model parameters (Beaumont, 2010).

We developed a robust, adaptive ABC scheme for infectious disease epidemiological data. This approach incorporates a parameter-free method of estimating the distribution of the data and includes an adaptive scheme for selecting tolerance values. We have developed this scheme as an open-source python library with examples demonstrating its use. In the first section of this paper, we directly compare ABC to a more standard Bayesian fitting technique as an example of where the likelihood is known, by modelling counts drawn from a negative-binomial distribution. We vary the heterogeneity (shape parameter) in the distribution to investigate how the fitting performs for different degrees of heterogeneity. We compare how well fitting performs as the number of tolerance levels and number of particles (parameter sets) changes, showing how the automated tolerance selection procedure produces accurate model fits. In the next section we apply the technique to two simple individual-based models, which include overly-dispersed one-dimensional data and two-dimensional time-series data respectively. The results show that this technique is amenable to a wide range of models and data with little coding overhead or hyper-parameter tuning. Finally we demonstrate the technique on a complex individual-based model of LF and show how disparate forms of data can be included in the model fitting process, highlighting the ease of incorporating multiple data sources into the fitting (Smith et al., 2017).

## 2 Methods

### 2.1 Epidemiological count data

Count data such as number of diagnosed cases in one year or parasite/viral load per patient are abundant in epidemiology. Often these data will be treated as being drawn from a Poisson distribution (Wakefield, 2007; Pullan et al., 2012). This is where the data is drawn from a probability distribution of the form

(1) $$P(X=x|\lambda )=\frac{\lambda^{x}}{x!}e^{-\lambda }.$$

The Poisson distribution is special because the mean and the variance are equal. Whilst there is some theoretical justification for this, often sources of data can be more over-dispersed, where the variance of the distribution is greater than the mean. In this case the data can be described as a negative-binomial. The issue is then how to measure the amount of over-dispersion. Techniques will often focus on a particular distribution such as maximum likelihood or Bayesian Markov chain Monte Carlo (MCMC). These techniques have proved highly effective for models where the underlying rates (such as those produced from deterministic differential equation models) can be described. Individual-based and other stochastic complex models are not amenable to this technique, however, and so approximate fitting methods have been considered, such as ABC. It is not clear, however, how to incorporate an appropriate goodness of fit metric for over-dispersed data (for example comparing the means would not be able to capture the heterogeneity in the distribution). Here we propose the use of kernel density estimation in order to resolve this problem.

Kernel density estimation (KDE) is a non-parametric scheme for approximating a distribution using a series of kernels, or distributions (Bishop, 2006). The technique has previously been applied to approximating the likelihood of a summary statistic (Fearnhead and Prangle, 2012; Gutmann et al., 2016). However we use it here to directly compare between the modelled and real data. An important benefit of this approach is that, unlike with histograms, where placement of bins is important, kernels are centred on each data point and hence bins do not need to be selected. Often a Gaussian kernel is chosen to represent the data, this has the useful property of allowing the distribution to be defined everywhere in parameter space, thus making it possible to compare two empirical distributions. Without this property, the methodology would be unable to compare between two different empirical distributions if there was not significant overlap.

### 2.2 Overview of ABC methodology

ABC is a technique used to perform Bayesian inference when a likelihood is either computationally intractable or not feasible to define. As an alternative, a sufficient summary statistic is used for the model data and compared to the data to be fitted. A distance metric is used to define the error between the data drawn from the model and the real data. As the error between the summary statistics of the model-generated data and real data approaches zero, the posterior distribution is approximated with greater accuracy (Csilléry et al., 2010; Beaumont, 2010; Kypraios et al., 2016).

More precisely, the function f summarises the data D in some form, for example, the mean parasite load in certain age-groups. For particular model parameters, θ, the model produces output $M_{\theta }^{*}$, where the star denotes this is a realisation of the model-data and is subsequently a random variable. We then define a distance metric, ρ, which compares the summary statistic from the data, f(D) with that from the model, $f(M_{\theta }^{*})$. The posterior is then approximated as the probability that the distance metric is below a threshold, ϵ, expressed as

(2) $$P(\theta |D)\approx P(\rho (f(D),f(M_{\theta }^{*}))<\epsilon ).$$

The error in the approximation is assumed to decrease as the threshold, ϵ, decreases, with the method being exact when the threshold is zero (Rubin et al., 1984). This approximation is dependent on the choice of summary statistic *f* and distance metric ρ, which are often problem-specific. The approximation also requires an appropriate choice of ϵ to increase accuracy and decrease computation time. If ϵ is too large then the drawn samples are often a poor approximation of the posterior, and if ϵ is too low, then only very rarely would sampled $M_{\theta }^{*}$ meet the criterion leading to increased computation time.

One of the simplest conceptual algorithms for ABC is a partial rejection scheme where a particle (parameter set) θ is drawn from the prior distribution Θ. This particle is then used in the model M to produce some sample data $M_{\theta }^{*}$. The sample data $M_{\theta }^{*}$ is then compared to the data *D* using the distance function ρ that gives a single-value for the discrepancy between the model data and the real data. This particle θ is then accepted if this discrepancy is below a pre-defined tolerance ϵ and rejected otherwise (Wilkinson, 2013) (e.g. for its first use see Pritchard et al., 1999, and see Blum and Tran (2010) for a smoothed rejection scheme applied to fitting an SIR model). In reality, this scheme can be inefficient if the prior is not similar to the posterior meaning that many particles are rejected. Also if the tolerance is too large then the sample of particles will be closer to the prior than the posterior. This means the scheme needs to be fine-tuned and may be impractical for most cases.

**Fig. 1:**
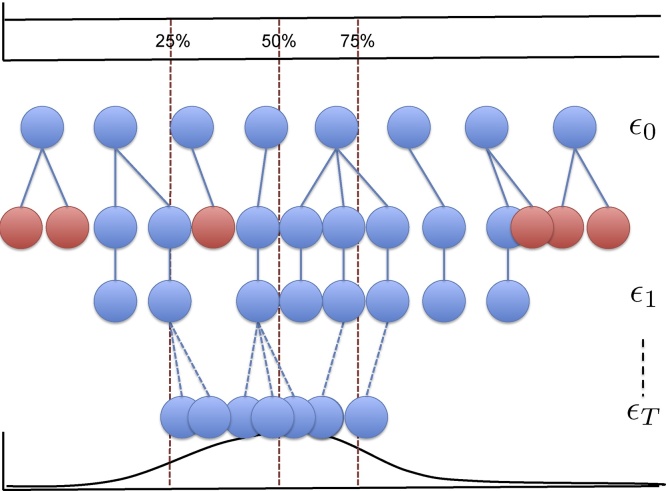
An overview of the ABC partial rejection control technique fitting to one parameter and for three steps. The true underlying likelihood is shown at the bottom, with 25, 50 and 75 percentiles shown as red dotted lines. A number of particles (*N*) are fixed at the beginning, here N=8. In the first step, these particles are drawn from a prior distribution, which is uniform between two values (top row, corresponds to steps 1–2 in Algorithm 1). For a given tolerance, a new particle is drawn for the updated tolerance $\epsilon_{1}$ by choosing a particle at random, perturbing it slightly and then running a model evaluation (step 5–6 in Algorithm 1). It then checks if the tolerance of that particle is below $\epsilon_{1}$, the particle is then either accepted (blue) or rejected (shown in red) (steps 7–8 in Algorithm 1). This procedure continues until all *N* particles are accepted at the new tolerance level (steps 9–10). As the tolerance decreases, the particles converge onto the target distribution (shown in the bottom row). (For interpretation of the references to color in text/this figure legend, the reader is referred to the web version of the article.)

A way of overcoming the low particle acceptance rate issue is to start with a large tolerance ϵ and then to proceed as above until the desired number of particles are selected (Fig. 1). These particles can then be used to generate an empirical distribution that can then replace the prior in the algorithm. The tolerance can then be lowered and the rejection scheme can be repeated until the desired number of particles are sampled. This scheme provides a way of lowering the tolerance to increase the accuracy, whilst also overcoming the issue of a small acceptance rate (Walker et al., 2010). The distribution of tolerances will depend heavily on the number of particles used, here we explore how the number of particles affects the final distribution (see supplementary material).

The challenges with this scheme are to choose a set of tolerances, $\left\{\epsilon_{t}\right\}$, to efficiently reduce the error in the samples. Typically a set is chosen prior to fitting. We considered two schemes for tolerance selection. The first is to generate a set of tolerances by sampling the prior distribution (Faisal et al., 2013). By drawing two sample particles $\theta_{1},\theta_{2}∼\Theta$ and recording the error between them $\epsilon =\rho (f(M_{\theta_{1}}^{*}),f(M_{\theta_{2}}^{*}))$, a distribution of error values can be built up from the prior distribution. A range of tolerances then may be chosen by taking the 0th, 10th, 20th, …, 100th percentile values of the error value distribution.

An alternative way of selecting tolerances is to do it adaptively, based on the distribution of errors that were accepted in the previous iteration (Beaumont et al., 2002). This is accomplished by recording for particle *i*, the accepted error $\tau_{i}$. The tolerance in the next iteration can then be chosen as some percentile of these values. Here, we adapted a scheme where the 50th percentile of these values was set as the new tolerance in order to keep the acceptance rate at reasonable levels. We found the adaptive scheme consistently outperformed the prior distribution scheme and as such we only consider the adaptive scheme here.

We considered data derived from both one-dimensional and two-dimensional distributions. The particular form of the summary statistic chosen for all examples was an empirical distribution derived from count data. Certain summary statistics and distance metrics such a the mean squared error between time-series data have underlying assumptions of normality and unimodality (Walker et al., 2010; Brown et al., 2018). We instead, adopt a scheme that is capable of incorporating a general distribution by using a non-parametric method to approximate the underlying probability density function *f* of the data. Note that here, as a simplification, discrete distributions are approximated by a continuous distribution. This was achieved using a Gaussian kernel-density estimator for the distribution. An empirical distribution $\hat{f}$ from count data $\left\{y_{i}\right\}$ was produced using a Gaussian kernel K by

(3) $$\hat{f}(x)=\frac{1}{n}\sum_{i=0}^{n-1}K(x-y_{i}).$$

Although each data-point is represented as a Gaussian with a small variance, the total distribution does not need to have the same properties and can for instance have higher variance or be multi-modal (Silverman, 1986). In order to compare between the two approximated distributions the non-symmetric Kullback–Leibler (KL) divergence was used. This measures the difference between the KDE-approximated probability distribution derived from the model data $\hat{p}$ and the KDE-approximated probability distribution derived from the real data $\hat{q}$. It is defined as

(4) $$D_{\mathrm{KL}}\left(\hat{p}||\hat{q}\right)=\int_{-\infty }^{\infty }\hat{p}(x)\log\frac{\hat{p}(x)}{\hat{q}(x)}dx,$$

where the divergence is greater than zero if the probability distributions differ and is zero if the distributions are equivalent. This method can also be easily adapted to a multivariate distribution, where an n-dimensional symmetric Gaussian with a fixed variance in each dimension can be used in the KDE step. The calculation of the KL divergence can also be extended by integrating over the entire support of the probability density function derived in the KDE step.

Our combined adaptive scheduling partial rejection control with kernel density estimation algorithm is as follows (Fig. 1). A number of particles (parameter sets) are drawn from the prior distribution P(θ) to produce a set of particles $\left\{\theta_{i}^{1}\right\}$. An initial tolerance value $\epsilon_{1}$ is found by selecting the median value of the KDE KL divergence between the data and model derived data from the selected particles. A new set of particles is generated by randomly sampling from $\left\{\theta_{i}^{1}\right\}$ and perturbed using a zero-mean Gaussian random variable with small variance. The newly generated particle is accepted if $D_{\mathrm{KL}}\left(f(D)||f\left(M_{\theta_{i}^{1}}^{*}\right)\right)<\epsilon_{1}$, else it is rejected and another particle is generated according the procedure defined. Once the desired number of particles have been accepted the tolerance is lowered adaptively by selecting the median value of the accepted tolerances from the previous iteration. A new set of particles is then generated as before with the lowered tolerance ϵ. Once the particles are generated for the smallest tolerance, $\epsilon_{T}$, the algorithm terminates and these are used as the sample for the posterior. A summary is given in Algorithm 1. We also show for a Gaussian likelihood that the minimization of the KL divergence with a KDE representation of the data is equivalent to maximising the likelihood (see supplementary material).

Algorithm 1 Adaptive ABC partial rejection control.

**Table:** 

### 2.3 Example applications of the method

#### 2.3.1 Example one: negative binomial distribution

As a first example in order to compare how fitting using our ABC scheme compares to other fitting techniques, samples were drawn from a negative binomial distribution with varying mean and heterogeneity parameter *k*. When k<1, the distribution is over-dispersed, with a greater variance to mean ratio than expected under a Poisson distribution. This means that the distribution is more heavy-tailed than for an equivalent Poisson distribution. When k>5, the distribution is less over-dispersed and small samples more closely resemble a Poisson distribution. In order to test how the parameter fitting performs for increasing heterogeneity (decreasing *k*), a sample is drawn from a negative-binomial parameterised by the mean *m* and heterogeneity *k*,

(5) $$P(X=x|m,k)=\frac{\Gamma (k+x)}{x!\Gamma (k)}{\left(\frac{k}{k+m}\right)}^{k}{\left(\frac{m}{k+m}\right)}^{x}.$$

The likelihood for an independent and identically distributed sample $X=(x_{0},x_{1},\ldots ,x_{n-1})$ is then

(6) $$P(X|m,k)=\prod_{i=0}^{n-1}\frac{\Gamma (k+x_{i})}{x_{i}!\Gamma (k)}{\left(\frac{k}{k+m}\right)}^{k}{\left(\frac{m}{k+m}\right)}^{x_{i}}.$$

*m* was varied between 1 and 100 and *k* was varied between 0.1 and 5. In order to be consistent between the samples the prior used in ABC was fixed for all samples before observing the data. Exponential priors were used with means of the distributions chosen to be the average of the ranges explored for *m* and *k*.

A Metropolis–Hastings MCMC scheme was also implemented and fitted to the negative-binomial count data (Gilks et al., 1995). The same priors that were used for the ABC scheme were also used for the MCMC scheme to provide a faithful comparison.

The impact of number of particles and size of tolerance were also explored using this model. For fixed parameters (m=50, k=3.0) the derived distribution was estimated for tolerances from 1 to 25 and particle numbers 10 to 200. The resulting estimated posterior was then compared to the true posterior (derived from the MCMC scheme).

The previous example can be easily implemented in the developed Python library with code that sets up a function that outputs an array of samples drawn from a negative binomial distribution for inputs *m* and *k* (denoted ibm), defines the priors as a list of functions that generate a sample for each parameter (denoted priors), provides the fitting object with the individual-based model, the data (denoted xs), the priors, and sets the method and number of steps to iterate through (denoted by the method setup) (Listing 1). The method is then run with a specified number of particles (denoted by the method run).

Listing 1 Code for negative binomial distribution example.

**Table:** 

#### 2.3.2 Example two: parasite model

As a simple epidemiological example, we propose an individual-based model where individuals acquire parasites at a constant rate that is drawn from a gamma-distribution with mean λ and shape parameter *k*. Each parasite within individuals are lost at a constant rate δ. When *k* is low the distribution of parasites is more heterogeneous with many individuals uninfected, but with a few highly infected, with very large parasite numbers. Schematically, the parasite dynamics within an individual $P_{i}$ can be written for each individual i;

(7) $$P_{i}\to P_{i}+1\;\mathrm{at}\;\mathrm{rate}\;\lambda b_{i},$$

(8) $$P_{i}\to P_{i}-1\;\mathrm{at}\;\mathrm{rate}\;\delta P_{i},$$

where $b_{i}$ is a random variable drawn from a gamma distribution with shape k and mean 1. This model could easily be extended by making the force of infection dependent on the current distribution of parasites as well as other factors such as environmental heterogeneity. It is however, meant to be instructive and as such the simplest form was used.

#### 2.3.3 Example three: stochastic SIS model

The stochastic Susceptible-Infected-Susceptible (SIS) model was implemented as an example of time-series data that can be estimated using a two-dimensional distribution approach. The model can be described as a Markov Process with two events: an infection and a recovery. For a population of size n, with the number of infected I, the infection and recovery events occur according to

(9) I→I+1atrateβ(n−I)I,

(10) I→I−1atrateγI.

The parameters β and γ can be reparameterised using the basic reproduction number $R_{0}$ and the expected time to recovery $\gamma^{-1}$ as $\beta =R_{0}/\gamma^{-1}$ and $\gamma =1/\gamma^{-1}$. The model was simulated in discrete time-steps using a tau-leaping algorithm and the corresponding likelihood was calculated using the corresponding transition rate matrix for the Markov Process (see supplementary material). In order to utilize this data with the KDE approach described we may convert the one-dimensional time-series data into two-dimensional distribution data in the following way, where we can explicitly take advantage of the Markov property of the underlying model. For a time-series of number of infected individuals recorded at regular intervals $\text{I}=(I_{0},I_{1},\ldots ,I_{T})$ the number of infected conditioned on the previous time-step $I_{t+1}|I_{t}$ can be represented as the matrix

(11) $$I_{t+1}|I_{t}={\left(\begin{array}{cccc} I_{0} & I_{1} & \ldots & I_{T-1}\\ I_{1} & I_{2} & \ldots & I_{T} \end{array}\right)}^{⊤}.$$

Each row in the matrix is a $\left(I_{t+1},I_{t}\right)$ pair which are points in 2D and can therefore be used to build up a two-dimensional probability density function (an example of this is shown in Fig. 3b).

With the given data representation the methodology is implemented in the Python package in exactly the same way as for the one-dimensional negative-binomial example. The output of the model function (ibm) is used to determine the dimension of the data and the list of prior random variable generators are used to determine the size of the parameter space in the model code.

#### 2.3.4 Example four: lymphatic filariasis

We used a stochastic individual-based model of lymphatic filariasis (Irvine et al., 2015). The model is a multi-scale stochastic simulation of individuals with worm burden, microfilaraemia (prevalence of the pre-larval stage of LF in the peripheral blood) and other demographic parameters relating to age and risk of exposure. Humans are modelled individually, with their own male and female worm burden denoted $W_{i}^{m}$ and $W_{i}^{f}$. The density of microfilariae (mf) in the peripheral blood is also modelled for each individual and denoted $M_{i}$. The total mf density in the population contributes towards the current density of L3 larvae in the human-biting mosquito population. The model dynamics are divided into the individual human dynamics, including age and turnover; worm dynamics inside the host; microfilariae dynamics inside the host and larvae dynamics inside the mosquito.

Five villages in the East Sepik Province of Papua New Guinea have been the focus of extensive research into filariasis epidemiology and transmission (Bockarie et al., 2003, Bockarie et al., 1998, Michael and Singh, 2016, Irvine et al., 2018). These villages received annual mass drug administration from 1993 through 1998, with no further interventions until bed-nets (LLIN) were distributed in August 2009. Self reported LLIN use ranged from 75% to 90% (Reimer et al., 2013).

Microfilaria prevalence were measured in these communities in 2008 as part of the post-MDA evaluation (Reimer et al., 2013). This was done by a BinaxNow filariasis antigen test and by microscopic evaluation of 1 mL filtered venous blood, collected at night. The age of participants was also recorded.

The KDE ABC methodology was implemented on three geographically variable parameters, the vector to host ratio *V/H*, the heterogeneity of bites *k* and the probability of an infective bite leading to an establishment of an adult worm s_2_. Each of these parameters were fit separately to the mf count distribution for each village. This was then compared to when the age-prevalence data was also included in the model fitting. Age-prevalence data was incorporated through the use of a mean squared distance function in addition to the KDE KL divergence function for the mf count data.

### 2.4 Implementation

The methodology and models were implemented in Python 2.7 (Python Software and Foundation, 2018), using the packages SciPy & NumPy (Van Der Walt et al., 2011) and seaborn for data visualisation (Waskom et al., 2014). An open-source python library, including examples can be found at the following URL: https://github.com/sempwn/ABCPRC. This library has been tested for both Python version 2.7 and version 3.6.

## 3 Results

### 3.1 Drawing from a negative binomial distribution

**Fig. 2:**
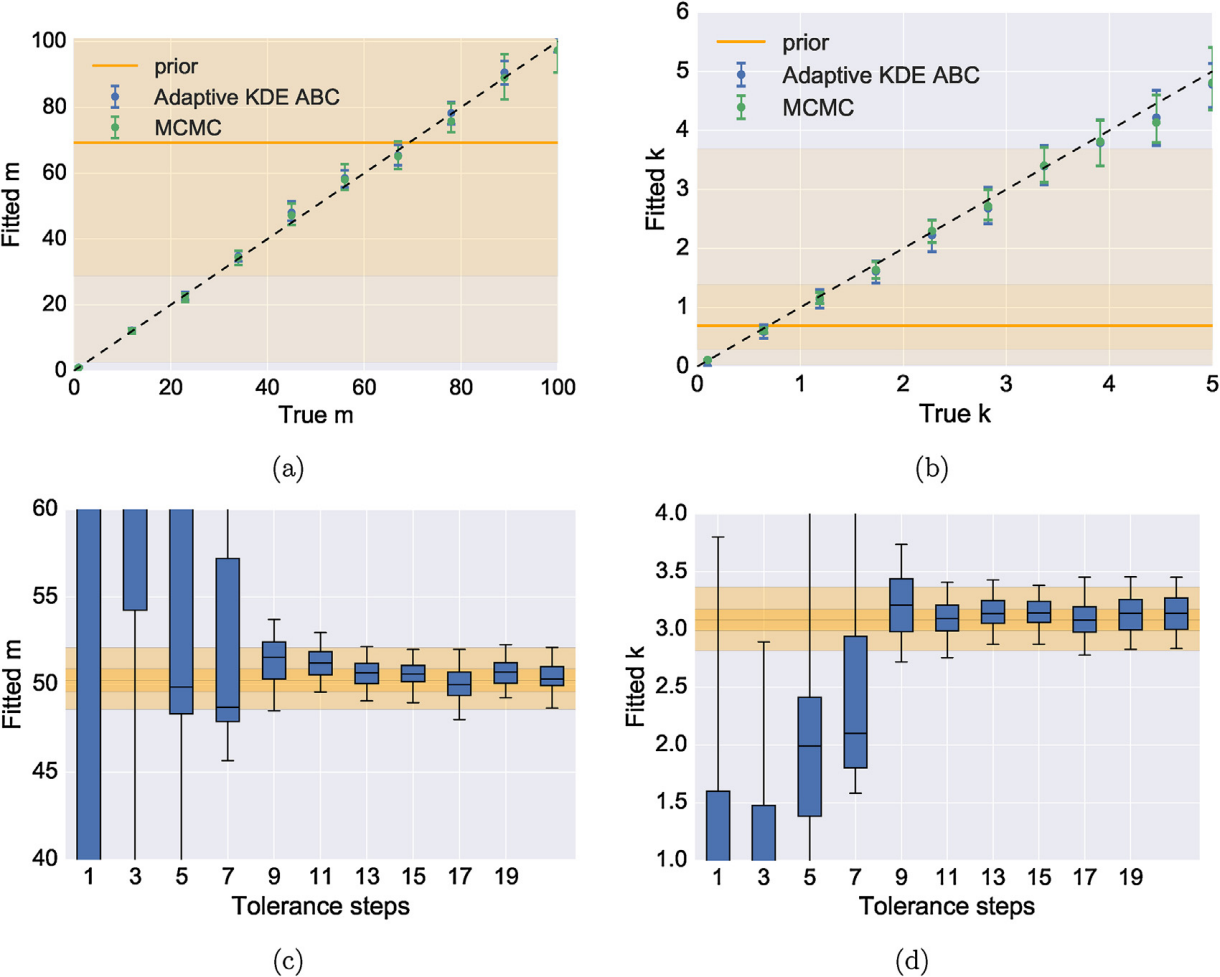
Comparison between MCMC and ABC methods for fitting a negative binomial distribution for a range of mean, *m* and heterogeneity *k*. (a) Comparison between fits for different mean values *m*, the dashed line represents the true values and the shading represents the 95% and 50% percentile range of the prior distribution, with the median given as a solid line. The prior distribution was kept fixed for each fitting. The adaptive KDE scheme closely matches the MCMC scheme for all values considered. When the resulting fit is biased for ABC, it is also biased in the same way for MCMC providing confidence that the scheme is approximating the true posterior. (b) Comparison between MCMC and the adaptive ABC scheme for heterogeneity *k*. As k>3 both the MCMC scheme and adaptive ABC scheme underestimate the true value in a consistent way due to the influence of the prior. Comparison between fitted distributions of the adaptive ABC scheme against the number of adaptive tolerance steps are shown for (c) *m* and (d) *k*. The true posterior calculated using MCMC is represented as a series of shaded regions with the 95% credible interval, 50% credible interval, and the median shown from lightest to darkest respectively. (For interpretation of the references to color in text/this figure legend, the reader is referred to the web version of the article.)

MCMC was directly compared to the adaptive ABC method using samples drawn from a negative binomial distribution with a range of means *m* and heterogeneities *k* (Fig. 2). The ABC scheme was ran on 100 particles over 25 tolerance steps, while the MCMC scheme was ran for 10,000 steps with a burn-in period of 2000 steps and a fixed step-size. Visual inspection was used to determine the convergence of the MCMC chains. Exponential priors with rate 50 and 1 were used for *m* and *k* respectively. For small *k*, samples from the distribution are more over-dispersed and larger *k* values more closely approximate the Poisson distribution. For all mean *m* values considered both the MCMC method and ABC method closely match the true value (Fig. 2a). As the size of *m* grows so does the size of the 95% credible interval in both cases. Where the model fit is biased due to the data realisation producing more than expected lower probability samples (e.g. mean value 50), both MCMC and ABC are biased in a consistent way. This provides more confidence that the scheme is recovering the true posterior distribution. Further evidence of this can be seen in the fitting as heterogeneity *k* varies (Fig. 2b). Here the prior is stronger, with a smaller 95% interval relative to the parameter range considered. For small values of *k*, the estimated posterior distributions closely match the true values. As *k* increases above 3 the true value moves outside of the prior's 95% range and thus begins to have more influence on the posterior. This can be seen as the expected value of *k* estimated from both methods is consistently lower than the true value.

**Fig. 3:**
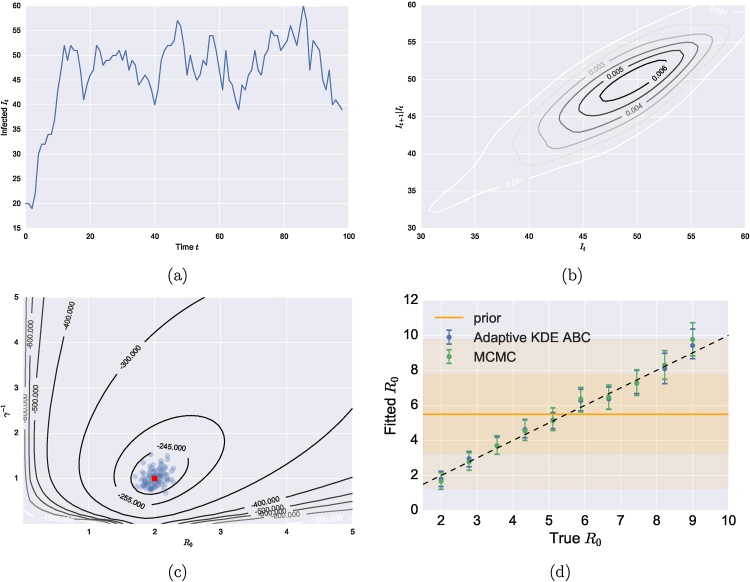
The Adaptive KDE ABC scheme for stochastic time-series data. (a) A realisation of the SIS process with $R_{0}=2$ population size n = 100 and recovery time $\gamma^{-1}=1$ (b) The corresponding time-series data converted into an empirical joint distribution using a two-dimensional KDE approach (level sets represent probability densities). (c) The estimated posterior distribution after 20 steps for 100 particles (in blue), with the true value in red. The true posterior is shown as level sets, with un-normalised log-values displayed. (d) Comparison of fitted distributions to *R*_0_ between the adaptive KDE approach against the MCMC method for a range of *R*_0_ values. The prior is represented as a series of shaded regions with the 95%, 50% and the median shown from lightest to darkest respectively. (For interpretation of the references to color in text/this figure legend, the reader is referred to the web version of the article.)

The number of adaptive tolerance steps strongly influences the estimated posterior for both the mean m (Fig. 2c) and the heterogeneity *k* (Fig. 2d). For a small number of steps (1-5), the estimate more closely resembles the prior distribution than the posterior distribution. From 7 to 9 steps the estimate is a combination of the prior and posterior distribution. For values above 10, the distributions closely match the true posterior. It should be noted that these values would likely change depending on the model and data, although we have found that 20 or more tolerance steps is sufficient for the estimate to converge to the posterior for the examples considered here.

The number of particles was also considered in how this hyper-parameter impacts the estimated posterior. Neither the expected value or the range were consistently effected by the particle size and even a small number of particles could approximate the true posterior reasonably well (see supplementary material). This suggests that if model evaluations are costly, then a small number of particles can be used to approximately determine the posterior before running the method on a large particle size.

### 3.2 Host–parasite model

The method was applied to the simple individual-based model of parasitic infection. A data sample was produced from the model (parameters: λ=10, δ=0.5, γ=1.0) and used in the fitting procedure. All parameters were given exponential prior distributions with mean rates broad enough to capture most dynamics. As the tolerance reduced, the variance in each of the marginal distributions lowered. The final distribution was unimodal for each parameter, with modal values close to the true underlying values. The final distribution also captures correlations between certain parameters such as between mortality and infection rate (see Fig. 2, supplementary material).

### 3.3 SIS model

A realisation of the SIS model was taken with parameters $R_{0}=2$, population size n = 100, and recovery time $\gamma^{-1}=1$, for 100 time-steps (Fig. 3a). The corresponding joint distribution of the $I_{t+1}$,$I_{t}$ data was approximated using a two-dimensional KDE (Fig. 3b). Here the joint distribution was approximately a bivariate correlated Gaussian, where the number of infected at time t+1 was strongly dependent on the number of infected at time t. The empirical distribution also has a longer tail than expected for a Gaussian distribution due to the initial transient phase where the infected population is rapidly increasing from the initial conditions. The adaptive KDE ABC method was able to accurately determine the correct *R*_0_ and $\gamma^{-1}$ values and was consistent with the true posterior (Fig. 3c). For other *R*_0_ values the adaptive ABC method was able to accurately approximate the true posterior and recover the true value (Fig. 3d).

### 3.4 LF in Papua New Guinea

**Fig. 4:**
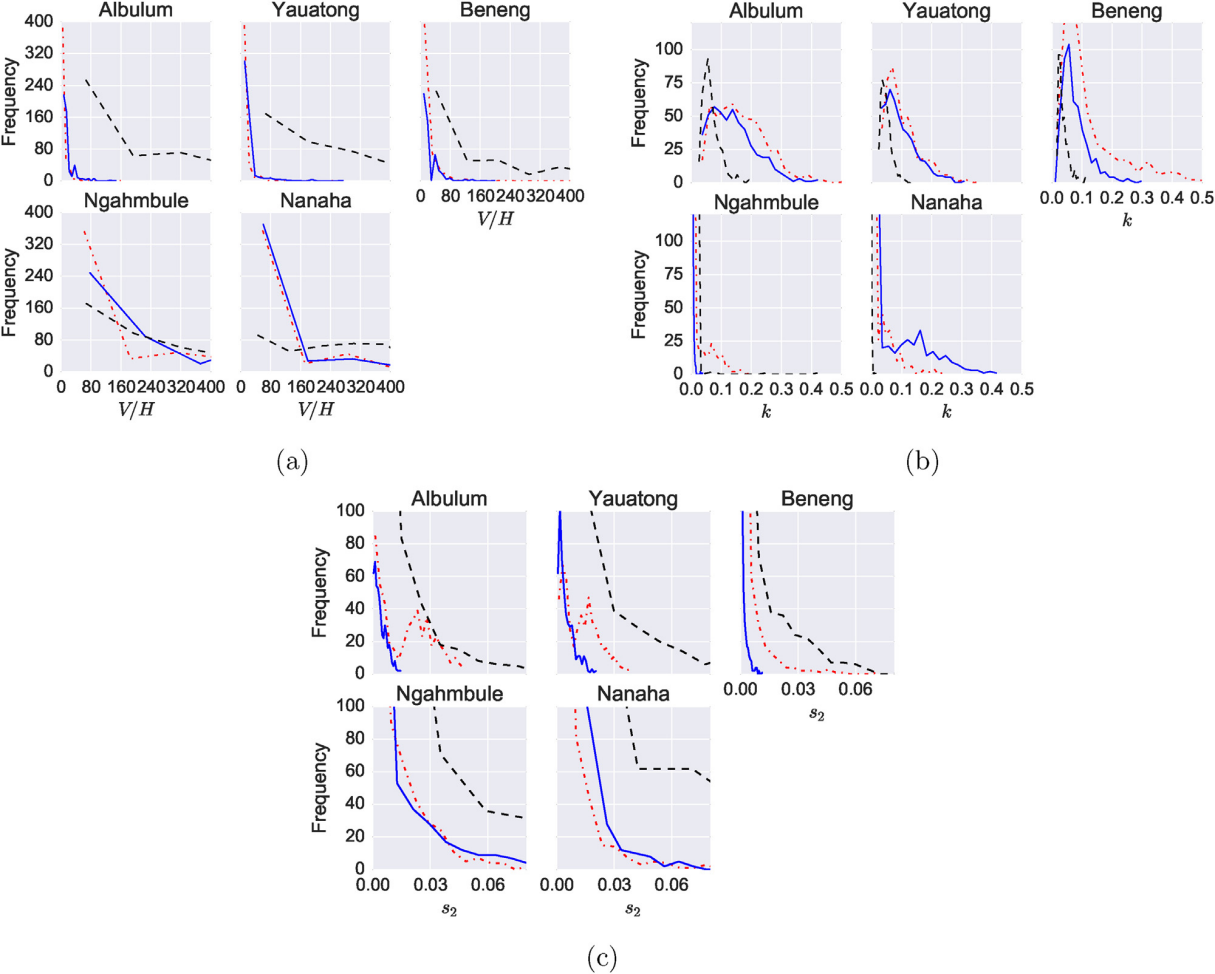
Results from fitting an individual-based lymphatic filariasis infection model to PNG. The estimated marginal distributions for parameters (a) vector to host ratio, V/H; (b) heterogeneity of exposure, *k* and (c) probability of larvae developing to reproductive adult, *s*_2_. Fitting just using count data in red; fitting using age prevalence data alone in black; and fitting using both count data and prevalence data in blue. (For interpretation of the references to color in text/this figure legend, the reader is referred to the web version of the article.)

Fitting was performed on five separate datasets of lymphatic filariasis infection including individuals’ age and mf count. The summary statistic used was derived from mf count alone; mf age-prevalence and a combination of the two. Three parameters were fitted, where other parameters in the model were derived from literature estimates. Age-prevalence alone was unable to accurately determine the vector to host ratio and the probability *s*_2_, with wide variances for the estimates of both (Fig. 4a and c). Using the mf count data only produced a smaller estimated range for these parameters, whilst giving a slightly wider range for the heterogeneity *k* (Fig. 4b). Combining both the mf count summary statistic and mf age-prevalence produces a more highly resolved marginal posterior for all three fitted parameters.

## 4 Discussion

Individual based models abound in epidemiology due to their intuitive description and greater ease of simulating many complex aspects of a system compared to deterministic models (Auchincloss and Diez Roux, 2008). These models increasingly involve processes that may not be easily captured by an ordinary differential equation or standard stochastic processes. This presents a great challenge, however, as standard fitting techniques have been developed for more traditional models, whereas ones for individual-based models have languished (Heesterbeek et al., 2015). Although for certain models it may be technically possible to write down a likelihood, there can be huge computational or technical barriers to do this. Whether this is due to a large number of hidden states or the sheer number of components in the model, this leads to having to resort to techniques such as visual inspection to perform fitting, introducing potential biases and not having a structured way to deal with the uncertainty in the fitted parameters. What is desirable is to have a technique where we can enjoy the benefits of Bayesian fitting, such as incorporating our prior knowledge and producing samples to estimate parameter uncertainty, without the often prohibitive procedure of conceiving of and calculating a likelihood.

Here we explored an ABC method as a solution for Bayesian model fitting. In particular we developed a technique that was amenable to a variety of data with minimal hyper-parameter tuning. The motivation is to provide a tool for model fitting with uncertainty quantification to a wide range of researchers, who may not have the necessary technical background to develop a full Bayesian approach with a developed likelihood. We performed model fitting using a summary statistic of the counts by approximating the distribution using a kernel density estimator. This allows fitting to be performed without explicit assumptions on the particular type of distribution the data takes as can be common with other model fitting techniques.

In order to compare the accuracy of ABC for increasingly heterogeneous count data, the procedure was carried out on various data generated from a negative-binomial distribution. For high heterogeneity, the procedure was able to accurately determine the shape parameter (*k*), as well as the mean parameter (*m*). This demonstrates that this technique is capable of handling a variety of heterogeneous data and can give similar results to standard Bayesian MCMC. the technique was also able to perform well on time-series data by transforming the data into a two-dimensional point representation. This technique would appear generally applicable to other time-series data including systems that may exhibit chaos (see supplementary material).

For many individual-based models a likelihood may be either computationally or analytically intractable. In these cases other methods have been proposed to overcome this issue. Using a partial rejection control scheme provides, at each iteration, a sample of particles (parameter sets) that are initially drawn from the prior, but as the tolerance decreases, these samples become more representative of the posterior. Although there are typically issues surrounding the choice of tolerances, such that the scheme is able to draw samples for the next iteration. Here, we overcome these issues by demonstrating two different schemes for choice a set of tolerances. This creates a much more efficient pipeline for fitting without the need to perform exploratory analysis of the error function beforehand (Walker et al., 2010).

One of the key issues with ABC is that it is an approximation method only. If the method does not sufficiently explore the space of parameters, the technique may produce spurious results. One possible diagnosis is to check the distribution of errors that were accept for each tolerance. If the errors are not significantly decreasing then this may indicate the procedure is stuck in a local minima and the variance of the priors may need increasing. The distribution of errors for the final tolerance can also indicate whether the procedure was halted prematurely or if lower tolerances can be accepted.

There is also an issue with the choice of summary statistics to be used and the number of parameters to fit to. It may be that some parameters can be estimated from independent studies, without the need to include them in the ABC procedure. It would then seem advisable to use these values either as a well-informed prior or as a point estimate as was done here. If the model is slow to evaluate, then this may also lead to practical fitting issues. Emulation methods may help to further increase the speed of fitting, by approximating the error manifold through the use of non-parametric fitting techniques such as Gaussian processes (Conti and O’Hagan, 2010; Drovandi et al., 2011).

One primary advantage of ABC over other techniques is the ability to utilize a range of data within model fitting. In the example of fitting an individual-based model of lymphatic filariasis infection to PNG data a combination of summary statistics was used. We explored fitting using just count data alone, constructing an empirical probability distribution and then comparing against the model count data using the KL divergence. This summary statistic was then combined with age-prevalence data, by constructing the prevalence in a defined set of age-categories and then using a weighted sum of squares in order to take into account the number of individuals in each age-category. We found that by adding in the extra information about the age-prevalence distribution the fitting was able to better resolve some of the parameters. ABC provides a way of incorporating many different types of data into the fitting and this suggests that the full number of pertinent summary statistics should be used.

## 5 Conclusion

The adaptive ABC method incorporating kernel density estimation and partial rejection control is a potentially powerful tool in model fitting for epidemiological data. We demonstrate that the same methodology can fit to both macro and micro-parasitic infectious diseases, one-dimensional or two-dimensional data, and can readily incorporate a wide array of data sources. In order for this tool to be readily-available to a wide-range of researchers we have developed this as an open-source python library, including example code to demonstrate its use.

## Data availability

All code is packaged as a python library and can be found at the following GitHub repository: https://github.com/sempwn/ABCPRC. This includes all code for generating data used in example model fitting.

## Competing interests

The authors declare no competing interests.

## Author contributions

The study was conceived by MAI & TDH. The coding, implementation, analysis and drafting of the manuscript was performed by MAI. All authors reviewed and approved the final manuscript. LF data are from the Papua New Guinea 2008 study (Reimer et al., 2013). The authors of this study may be contacted at jxk14@case.edu.

## Funding

The authors gratefully acknowledge funding of the NTD Modelling Consortium by the Bill and Melinda Gates Foundation in partnership with the Task Force for Global Health. The funder had no role in study design, data collection and analysis, decision to publish, or preparation of the manuscript. The views, opinions, assumptions or any other information set out in this article should not be attributed to Bill & Melinda Gates Foundation and The Task Force for Global Health or any person connected with them.
