# Supplementary material for: Kernel-density estimation and approximate Bayesian computation for flexible epidemiological model fitting in Python
Source: Epidemics. 2018 Dec;25:80–8. doi: 10.1016/j.epidem.2018.05.009 (PMC6227249; doi:10.1016/j.epidem.2018.05.009)
Supplement: Supplementary file 1 [file mmc1.pdf]

Kernel-density estimation and Approximate Bayesian Computation for flexible epidemiological model fitting in python: Supplementary information

Michael A Irvine*[1] and T Deirdre Hollingsworth†[1]

[1]School of Life Sciences, University of Warwick, UK

May 4, 2018

Derivation of KL-divergence for kernel density estimation

We wish to demonstrate the minimising the KL-divergence for a KDE representation of a distribution is asymptotically equivalent to the likelihood up to some constant. The likelihood for a normal distribution with data $\{d_i\}$ is

$$\prod_{i=1}^{n} \frac{1}{\sqrt{2\pi\sigma^2}} \exp(-\frac{(d_i-\mu)^2}{2\sigma^2}). \tag{1}$$

Equivalently, the log-likelihood l is

$$l = -\frac{n}{2}\log(2\pi\sigma^2) - \sum_{i=1}^{n} \frac{(d_i-\mu)^2}{2\sigma^2}. \tag{2}$$

We will now derive an asymptotic approximation of the KL-divergence for a Gaussian KDE representation of the data $\{d_i\}$. The empirical distribution $p(x)$ is defined using a series of Gaussian probability distribution with variance σ_K^2 as,

$$p(x) = \sum_{i=1}^{n} r_{\sigma_K}(x-d_i), \tag{3}$$

where $r_\sigma(x) = \frac{1}{\sqrt{2\pi\sigma^2}}\exp(-\frac{x^2}{2\sigma^2})$. The Kullback-Liebler Divergence between the true distribution $q(x)$ and the empirical distribution $p(x)$ is defined as

$$D_{KL}(P||Q) = \int_{-\infty}^{\infty} p(x)\log\left(\frac{p(x)}{q(x)}\right)\mathrm{d}x \tag{4}$$

Inputting Eq. 3 into Eq. 4 produces

$$D_{KL}(P||Q) = \int_{-\infty}^{\infty} \sum_{i=1}^{n} r_{\sigma_K}(x-d_i)\log\left(\frac{\sum_{i=1}^{n} r_{\sigma_K}(x-d_i)}{q(x)}\right)\mathrm{d}x. \tag{5}$$

*m.irvine@math.ubc.ca

†Deirdre.Hollingsworth@bdi.ox.ac.uk

Through manipulation of the right-hand side,

$$\begin{aligned}D_{KL}(P||Q) &= \int_{-\infty}^{\infty}\sum_{i=1}^{n} r_{\sigma_K}(x-d_i)\log\left(\frac{\sum_{i=1}^{n} r_{\sigma_K}(x-d_i)}{q(x)}\right)\mathrm{d}x,\\ &= \int_{-\infty}^{\infty}\left[\sum_{i=1}^{n} r_{\sigma_K}(x-d_i)\right]\log\left(\sum_{i=1}^{n} r_{\sigma_K}(x-d_i)\right)\mathrm{d}x - \int_{-\infty}^{\infty}\sum_{i=1}^{n} r_{\sigma_K}(x-d_i)\log\left(q(x)\right)\mathrm{d}x,\\ &= -H\left(\sum_{i=1}^{n} R_{\sigma_K}^{d_i}\right) - \sum_{i=1}^{n}\int_{-\infty}^{\infty} r_{\sigma_K}(x-d_i)\log\left(q(x)\right)\mathrm{d}x. \end{aligned} \tag{6}$$

Where H is the differentiable entropy and R_{σ}^{m} is the normal random variate with mean m and variance σ^2. Using the relationship $H(X+Y) \le H(X)+H(Y)$ and the equation for the integral between two univariate normal distributions is $\int_{-\infty}^{\infty} r_{\sigma_K}(x-d_i)\log\left(q(x)\right)\mathrm{d}x = -\frac{1}{2}\log(2\pi\sigma^2) - \frac{\sigma_K^2}{2\sigma^2} - \frac{(\mu-d_i)^2}{2\sigma^2}$,

$$\begin{aligned}D_{KL}(P||Q) &= -H\left(\sum_{i=1}^{n} R_{\sigma_K}^{d_i}\right) - \sum_{i=1}^{n}\int_{-\infty}^{\infty} r_{\sigma_K}(x-d_i)\log\left(q(x)\right)\mathrm{d}x,\\ &\ge -n\log(\sigma_K\sqrt{2\pi e}) - \sum_{i=1}^{n}\left(-\frac{1}{2}\log(2\pi\sigma^2) - \frac{\sigma_K^2}{2\sigma^2} - \frac{(\mu-d_i)^2}{2\sigma^2}\right),\\ &= -n\log(\sigma_K\sqrt{2\pi e}) + \frac{n}{2}\frac{\sigma_K^2}{\sigma^2} + \frac{n}{2}\log(2\pi\sigma^2) + \sum_{i=1}^{n}\frac{(\mu-d_i)^2}{2\sigma^2}.\end{aligned} \tag{7}$$

We hence have $D_{KL}(P||Q) \ge -l(\theta)$ and hence minimizing the KL divergence is equivalent to maximising the likelihood as required.

Derivation of likelihood for stochastic SIS model

We derive the likelihood for a stochastic SIS model where number of infected individuals are recorded at regular intervals. The SIS or susceptible-infected-susceptible model describes an infectious disease where no immunity is acquired after an infection and an individual becomes completely susceptible when the infection is cleared. It is also implicitly assumed that the population remains constant (i.e. there is no immigration or emigration). The rates may be written down as

$$S \to I \text{ at rate } \beta, \tag{8a}$$
$$I \to S \text{ at rate } \gamma, \tag{8b}$$
$$\tag{8c}$$

where β is the infection rate and γ is the recovery rate. This is an example of a birth-death process where both the birth and death rates are dependent on the population size. For a given population size N the mean field dynamics may be written as an ODE of the form

$$\frac{\mathrm{d}I}{\mathrm{d}t} = \frac{\beta}{N}S(I+\epsilon) - \gamma I. \tag{9}$$

or more compactly,

$$\frac{\mathrm{d}I}{\mathrm{d}t} = \frac{\beta}{N}(N-I)(I+\epsilon) - \gamma I. \tag{10}$$

ϵ is used to ensure that extinction of the disease cannot occur and is the importation rate of the pathogen. We may write down the equivalent Kolmogorov forward equation as

$$\frac{\mathrm{d}p_n}{\mathrm{d}t} = p_{n-1}\left[\frac{\beta}{N}(N-n+1)(n-1+\epsilon)\right] + p_{n+1}\left[\gamma(n+1)\right] - p_n\left[\frac{\beta}{N}(N-n)(n+\epsilon) + gn\right]. \tag{11}$$

More compactly this may be written as a linear ODE using the notation $\mathbf{p} = (p_0, \ldots, p_N)$ as,

$$\frac{\mathrm{d}\mathbf{p}}{\mathrm{dt}} = \mathbf{p}Q, \tag{12}$$

where Q is a matrix defined as,

$$Q = \begin{pmatrix} -\beta\epsilon & \beta\epsilon & 0 & 0 & \ldots & 0 \\ g & -(\beta(N-1)(1+\epsilon)/N + g) & \beta(N-1)(1+\epsilon)/N & 0 & \ldots & 0 \\ \vdots & \vdots & \vdots & \vdots & \ddots & \vdots \\ 0 & 0 & 0 & 0 & \ldots & -Ng \end{pmatrix}. \tag{13}$$

Solving Eq. 12 gives the probability of being in a given state at time t given a state at time 0,

$$\mathbf{p}(t) = \mathbf{p}(0)\exp(tQ) \tag{14}$$

For example if it is known that at time 0 there are n_0 infected individuals then the probability at time t is

$$\mathbf{p}(t) = \mathbb{I}[n_0]\exp(tQ). \tag{15}$$

This provides a way of calculating the likelihood for a set of parameters $\theta = (\beta, \gamma, \epsilon)$ and data $D = (i_k; t_k)$ for $k = 1, \ldots, m$.

$$l(\theta|D) = [p(\theta; t_1)]_{i_1} \prod_{k=2}^{m} [\exp((t_k - t_{k-1})Q)]_{i_{k-1}, i_k}. \tag{16}$$

For simplicity we assume that prevalence is recorded at regular intervals and all parameters are scaled such that this time-interval is one. The associated log-likelihood is then,

$$\log l(\theta|D) = \sum_{k=2}^{m} \log\left([\exp(Q)]_{i_{k-1}, i_k}\right). \tag{17}$$

Ricker Model

The Ricker model was constructed as an example of an ecological chaotic system [1]. The Ricker model is a discrete stochastic map, where the density of a population (N_t) is dependent on the previous time's density (N_{t-1}) with some noise term ϵ_t. The map has the following form

$$N_t = N_{t-1}\exp(-rN_t + \epsilon_t) \tag{18}$$

The noise term is drawn from a normal with zero mean and variance σ_e, i.e. $\epsilon_t \sim N(0, \sigma_e)$. r represents the density-dependent reduction in population growth, where a small r produces a stable fixed point, with larger r values leading to chaos. Finally the population density N_t is connected to the observed population size y_t by the Poisson distribution.

$$y_t \sim Poi(\phi N_t) \tag{19}$$

where ϕ is some observation factor. Previous methods have considered constructing a synthetic likelihood for this system [2]. Here we consider using the KDE-ABC scheme to determine the parameters (ϕ, σ_e, r). The fitting scheme was set up in similar fashion to the stochastic SIS model in the main text. Each observation y_t was used to construct a matrix of adjacent values,

$$y_{t+1}|y_t = \begin{pmatrix} y_0 & y_1 & \cdots & y_{T-1} \\ y_1 & y_2 & \cdots & y_T \end{pmatrix}^{\intercal}. \tag{20}$$

.

Data were generated from the model for 200 time-steps and with parameters $\log(r) = 2$,$\sigma_e = 1$, and $\phi = 10$, where the system is nearly chaotic. Uniform priors were chosen for each parameters with $\log(r) \sim U(0, 10)$, $\sigma_e \sim U(0, 5)$, and $\phi \sim U(0, 20)$. The KDE-ABC fitting scheme was ran with 1000 particles for 20 tolerance steps.

The results are given in Fig. 3. The KDE-ABC scheme was able to recover all three parameters and the sampled particles differed greatly from the priors indicating the scheme was strongly informing the estimated posterior.

References

[1] Peter Turchin. *Complex population dynamics: a theoretical/empirical synthesis*, volume 35. Princeton university press, 2003.

[2] Simon N Wood. Statistical inference for noisy nonlinear ecological dynamic systems. *Nature*, 466(7310):1102, 2010.

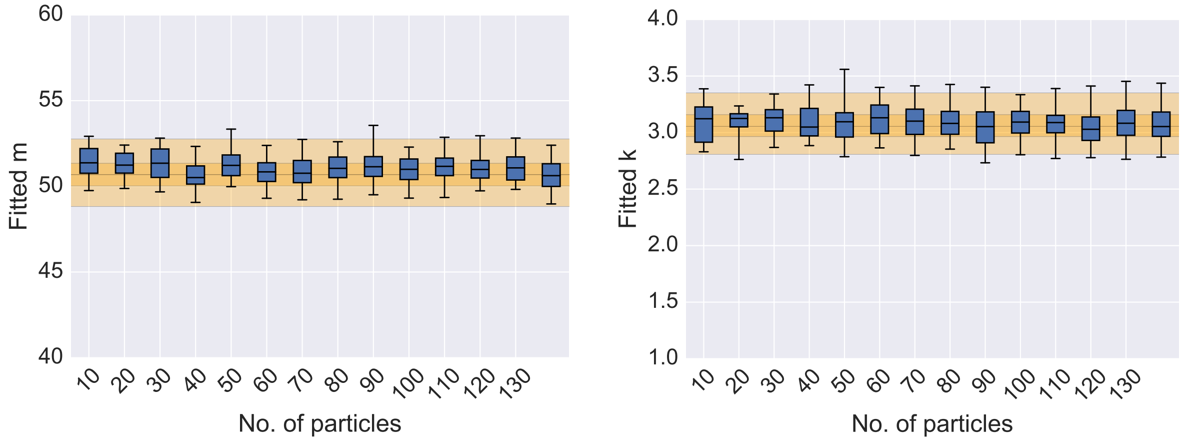

(a) (b)

Figure 1: Effect of number of particles on the posterior samples. The orange shading represents the median, inter-quartile range and 95% percentiles of the true posterior and the box-plots are the estimated posterior for each particle number. Results shown for **(a)** Estimated mean and **(b)** estimated shape parameter.

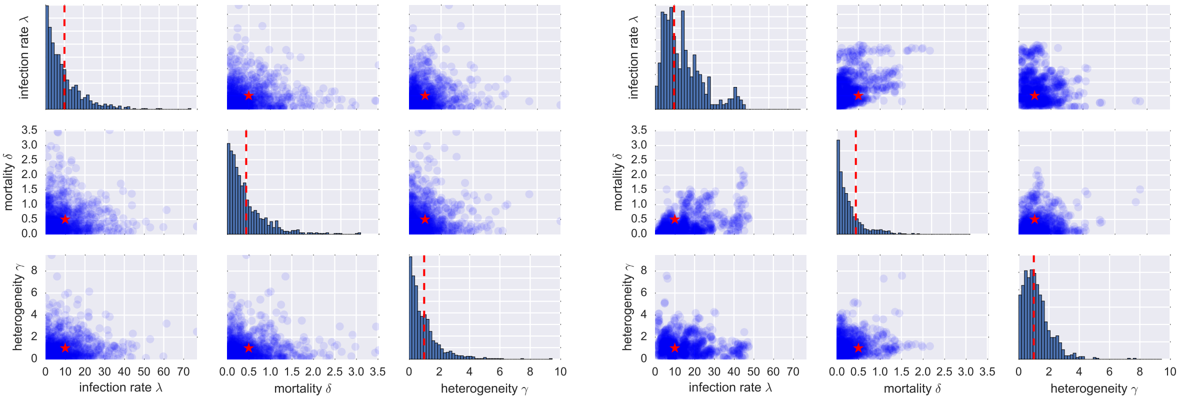

(a) (b)

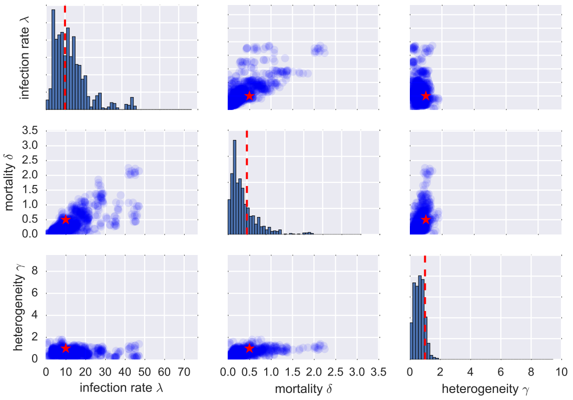

(c)

Figure 2: ABC fitting to simple epidemiological model of parasitic infection. The initial distribution of particles is shown in **(a)**, the distribution half-way through is shown in **(b)** and the final distribution is given in **(c)**. The true values $\lambda = 10$,$\delta = 0.5$ and $\gamma = 1.0$ are shown as red dotted lines in the marginal distributions and as a red asterix in the joint distributions.

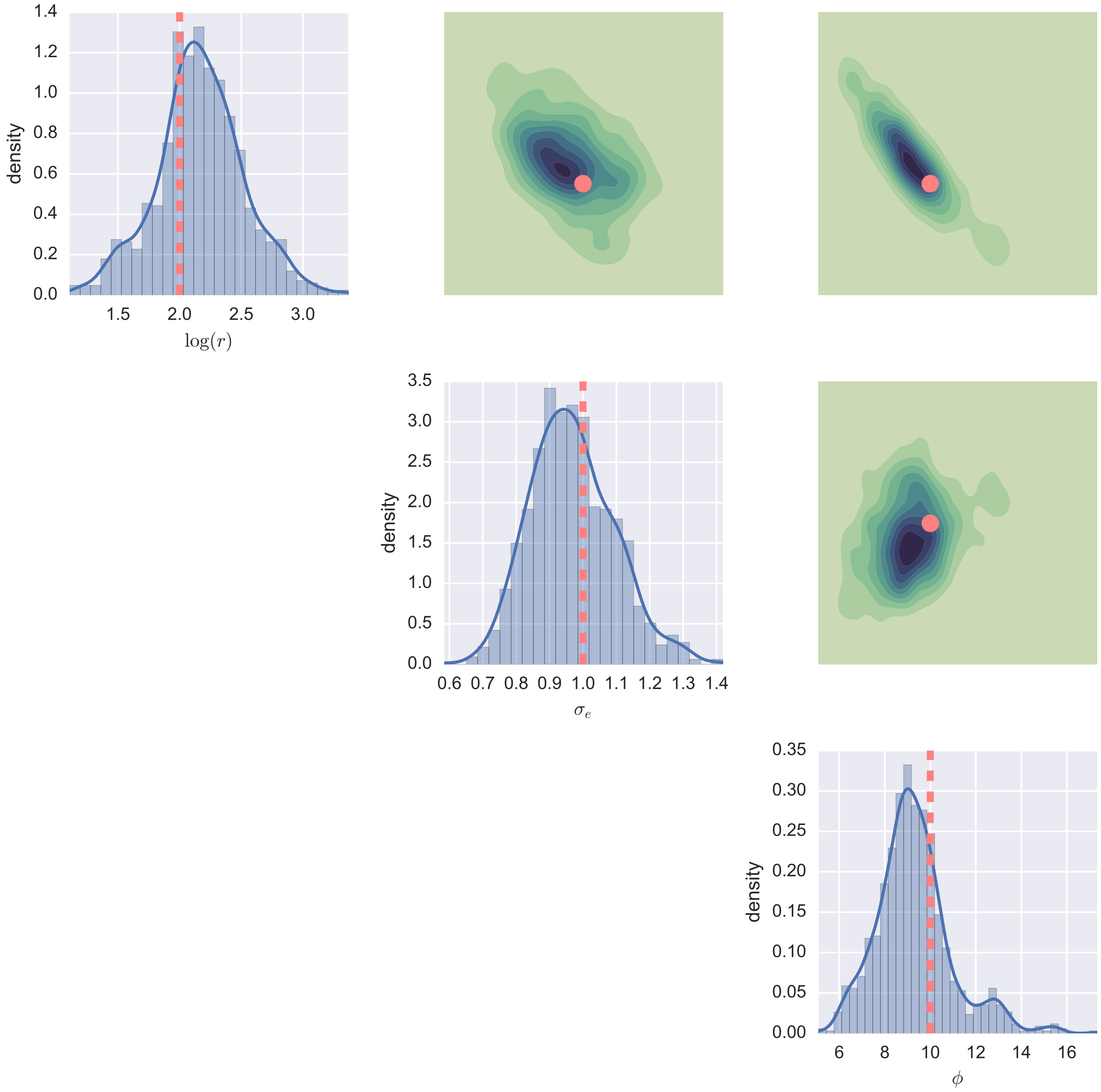

Figure 3: Example of fitting to the Ricker model. Figure shows the estimated posterior distribution derived from the particles sampled from the ABC-KDE scheme. The marginal distribution for parameters $\log(r)$, σ_e and ϕ are shown along the diagonal, with the pair-wise joint distributions shown as density contour plots (where density increases from light to dark). The true values used to generate the data are shown as red dashed lines in the marginal plots and as red points in the joint plots. All three parameters were recovered from the scheme.
